# Strategic Planning, Implementation, and Evaluation Processes in Hospital Systems: A Survey From Iran

**DOI:** 10.5539/gjhs.v7n2p56

**Published:** 2014-09-28

**Authors:** Jamil Sadeghifar, Mehdi Jafari, Shahram Tofighi, Hamid Ravaghi, Mohammad Reza Maleki

**Affiliations:** 1 Department of Health Services Management, School of Health Management and Information Sciences, Iran University of Medical Sciences, Tehran, Iran; 2 Health Management Research Centre, Baqiyatallah University of Medical Sciences, Tehran, Iran; 3 Health Management and Economics Research Center, Iran University of Medical Sciences, Tehran, Iran

**Keywords:** strategic planning, strategic implementation, strategic evaluation, strategic control, hospital, Iran

## Abstract

**Aim & Background:**

Strategic planning has been presented as an important management practice. However, evidence of its deployment in healthcare systems in low-income and middle-income countries (LMICs) is limited. This study investigated the strategic management process in Iranian hospitals.

**Methods:**

The present study was accomplished in 24 teaching hospitals in Tehran, Iran from September 2012 to March 2013. The data collection instrument was a questionnaire including 130 items. This questionnaire measured the situation of formulation, implementation, and evaluation of strategic plan as well as the requirements, facilitators, and its benefits in the studied hospitals.

**Results:**

All the investigated hospitals had a strategic plan. The obtained percentages for the items “the rate of the compliance to requirements” and “the quantity of planning facilitators” (68.75%), attention to the stakeholder participation in the planning (55.74%), attention to the planning components (62.22%), the status of evaluating strategic plan (59.94%) and the benefits of strategic planning for hospitals (65.15%) were in the medium limit. However, the status of implementation of the strategic plan (53.71%) was found to be weak. Significant statistical correlations were observed between the incentive for developing strategic plan and status of evaluating phase (*P*=0.04), and between status of implementation phase and having a documented strategic plan (*P*=0.03).

**Conclusion:**

According to the results, it seems that absence of appropriate internal incentive for formulating and implementing strategies led more hospitals to start formulation strategic planning in accordance with the legal requirements of Ministry of Health. Consequently, even though all the investigated hospital had the documented strategic plan, the plan has not been implemented efficiently and valid evaluation of results is yet to be achieved.

## 1. Introduction

In recent years, improving the quality of health services has attracted the attention of many scientific investigations (Arah et al., 2003). The phases that healthcare organizations routinely follow for improvement of services provision quality are setting priorities, establishing sustainable processes and determining an appropriate framework to implement the initiative programs (Glickman, Baggett, Krubert, Peterson, & Schulman, 2007; Rütten, Röger, Abu-Omar, & Frahsa, 2009). On the other hand, increasing global competition has led these organizations to re-engineering of business processes as a means to ensure efficiency, effectiveness and ultimately their success (Jafari, Bastani, Ibrahimipour, & Dehnavieh, 2012). One of the most effective strategies for the success of organizations is running the strategic planning. It is one of the most common managerial practices in healthcare provider organizations, largely in the form of research articles that have not been evaluated. Evidence-based management seems inevitable in these organizations to conduct investigations on such common practice (Kaissi, Begun, & Welson, 2008).

Strategic planning is the systematic process whereby an organization creates a document indicating the way it plans to progress from its current situation to the desired future situation (Perera & Peiró, 2012). According to Swayne, Duncan, and Ginter (2006), strategic planning is a set of processes, which help in identifying the future desired by the organization and to developing guidelines for making the decisions leading to such a future (Swayne, Duncan, & Ginter, 2006). Strategic planning integrates main objectives, policies and associated activities in an individual organization as a whole. A strategy based on the shortcomings and the competence within the enterprise can anticipate changes in the environment, and can be helpful for allocation of enterprise resources in a unique and valuable way (Mintzberg, Quinn, & Ghoshal, 1998).

Successful strategic planning necessitates development of the plan in right manner, as well as appropriate implementation, accurate and on-time evaluation of the results, possibly through the Strategic Control System (SCS). The SCS involves tracking strategies to action, identifying the problems or changes in necessary strategies and reforms. Senior manager must constantly ask himself/herself whether the organization is moving in the right direction or not, and if the assumptions about the intended major trends and changes in the environment are correct. Such questions need to set the strategic controls (Pearce & Robinson, 2007).

Evidence of strategic planning in low-income and middle-income countries (LMICs) is very low (El-Jardali, Jamal, Abdallah, & Kassak, 2007). Compared with high-income countries, healthcare environment in LMIC is more complex, dynamic and challenging (Mills, Brugha, Hanson, & McPake, 2002) where resources are generally more limited. Thus, the need for strategic planning in these countries should be understood to a larger degree. Saleh et al. (2013) in research on strategic planning processes and their relation to the financial performance of hospitals in Lebanon examined six dimensions including having a plan, plan development, plan implementation, responsibility of planning activities, governing board involvement, and physicians’ involvement as components of the strategic planning process (Saleh, Kaissi, Semaan, & Natafgi, 2013). In a research study, among hospitals in Texas, Kaissi et al. (2008) demonstrated that 87% of the hospitals had a strategic plan, and concluded that three dimensions including having a strategic plan, assigning the Chief Executive Officer (CEO) for the plan, and involving the board are positively associated with financial performance (Kaissi et al., 2008).

Iran as a LMIC is an ancient country located in the Middle East, a region between Asia, Europe, and Africa. The Iranian healthcare system is pluralistic and fragmented because of the public-private mixture in financing and provision of health services. At the national level, Ministry of Health is exercising the governance, policy-making, planning, financing and steering the programs. At the provincial level, the Universities of Medical Sciences and Health Services are responsible for provision of health services, including the environmental health (Mehrdad, 2009).

It should be noted that according to the Iranian national accreditation system (effective from 2010); hospitals are required to have a strategic plan that indicates future directions of the hospital (Jafari et al., 2010). Although having a strategic plan is an overarching national health initiative, this study went beyond the requirement of having a strategic plan to assess the actual process and identify the stakeholders involved in the strategic planning within hospitals. The goal of this study was to explore the process of formulation, implementation and evaluation of strategic plans in all the teaching hospitals affiliated of Iran and Tehran Universities of Medical Sciences.

## 2. Methodology

### 2.1 Sample

The research was conducted on the teaching hospitals affiliated with Tehran University of Medical Sciences (TUMS) and Iran University of Medical Sciences (IUMS) in Tehran (N = 26). There are three large medical universities in Tehran that cover medical services through governmental and teaching hospitals. The TUMS and IUMS have 17 and 10 teaching hospitals, respectively. Three hospitals refused to participate in the study; thus, the study was done on 24 hospitals. From the studied hospitals, a total of 8 hospitals were general and 16 hospitals were specialized.

### 2.2 Data Collection

The data gathering tool was a questionnaire developed by the authors based on the literature review (Kaissi et al., 2008; Poister, & Streib, 2005) and expert opinion. The questionnaire consisted of 130 questions and its validity was confirmed by seven experts in the field of strategic planning. The questionnaire included the following sections and questions:

*- Demographic variables (9 items)*: Specialty of hospital, number of bed, number of employees, hospital age, grade evaluation, field of study and education degree of hospital manager, the number and composition of the strategic planning committee.

*- Requirements and Facilitators of Strategic management (7 items)*: management systems deployed in hospital, using a consultant in strategic planning, having a strategic planning committee, activation of strategic planning committee, having a documented strategic plan, review of the strategic plan, and incentive for developing strategic plan.

*- The strategic planning phase*: extent of stakeholders’ contribution (chief of hospital, hospital manager, assistants and matron, clinical departments officials, officials of the supportive sections, staff at operational levels, external stakeholders, representatives of patient/donor, representative of university) in the strategic planning process in 5 dimensions including participation in meetings, provision of the required information, provision of the initial ideas, provision of reformed and completive suggestions for plan development, presentation of gathering and documentation of plan (45 items), and the extent of attention to various components of the strategic planning (15 items).

*- The strategic implementation phase*: the components of the budget allocation based on strategic priorities (6 items) and taking action based on the strategic plan (7 items).

*- The Strategic evaluation phase*: status of using indicators for achieving goals (15 items).

*- The benefits of the strategic planning* (25 items) and a question to determine the level of strategic planning usage in hospitals.

The scale used for the questions relating to the requirements and facilitator of strategic management was “yes/no”, with a range of weak (0-6), to moderate (7-9) and good (12-10). For the remaining questions, the 5-degree scale (5: very good to 1: very low) and triple range from weak (0-2.50), to moderate (2.51-4) and good (4.01-5) were used to analyze and interpret the answers. In order to unifying the scorings, the calculated scores in both the requirements and the strategic management process have been changed based on 100% and classified as following, Low: 0 to ≤ 50- Moderate: greater than 50% to ≤ 80 - Good: greater than 80%.

Data were collected by the researchers at the studied hospitals and the questionnaire was completed by the investigator in the form of interview. In each hospital, the key person/persons with the greatest involvement and awareness of the hospital’s strategic plan were interviewed. Job status of persons that participated in this study in the investigated hospitals included quality improvement officer (13 hospitals), hospital affairs expert (4 hospitals), clinical governance officer (2 hospitals), hospital manager, Research and Development executive (R&D), clinical supervisor, and systems and procedures officer. Objectives of the study were explained to the participants, and to confirm the accuracy of answers, the documents available in the hospitals were also studied if further review required.

### 2.3 Data Analysis

Data analysis was performed using the Statistical Package for the Social Sciences (SPSS) software, version 18.0. In this study, the strategic management requirements included 10 variables (defined in Table 2) as the independent variables, that all of them have a two-way scale. Furthermore, the strategic management process (including planning, implementation and evaluation) has been considered as the dependent variables. Therefore, in order to compare the mean of the differences between the dimensions of strategic management with against the requirements, we used the t-test (Table 4).

**Table 1:** Characteristics of hospitals and hospital managers

Variables		n	%
Type of hospital	General	7	29.2
	Specialized	17	70.8
	
Evaluation degree	Excellent degree	6	25
	One degree	18	75
	
Number of beds	Less than 200	12	50.0
	201 to 500	10	41.7
	More than 501	2	8.3
	
Hospital age	Less than 30	2	8.3
	31 to 50	9	37.5
	More than 51	13	54.2
	
Number of staff	Less than 500	15	62.5
	More than 501	9	37.5
	
Manager’s level of education	BSc.	2	8.3
	MSc.	11	45.8
	Professional Doctorate	4	16.7
	Ph.D.	7	29.2
	
Manager’s field of education	Health Services Management	14	58.3
	Others	10	41.7

**Table 2:** Situation of strategic management process requirements and facilitators

	Variables	n	%
Employing consultant	Yes	14	58.3
	No	10	41.7
	
Employing ISO 9001	Yes	7	29.2
	No	17	70.8
	
Employing EFQM	Yes	8	33.3
	No	16	66.7
	
Having a strategic planning committee	Yes	20	83.3
	No	4	16.7
	
Active strategic planning committee	Yes	12	60.0
	No	8	40.0
	
Strategic planning committee members	5 to 9	11	55.0
	More than 10	9	45.0
	
Having a documented strategic plan	Once	11	45.8
	More than once	13	54.2
	
Having revision in strategic plan	Yes	19	79.2
	No	5	20.8
	
Incentive for developing strategic planning	Legal driven	15	62.5
	Improvement driven	9	37.5
	
Employing Balanced Scorecard (BSC)	Yes	9	37.5
	No	15	62.5

**Table 3:** Situation of strategic planning process, requirements, facilitators and benefits

	Variables	n	%	Mean	SD
Requirements and facilitators of the strategic planning	Weak	4	16.7	68.75	15.59
	Moderate	15	62.5		
	Good	5	20.8		
	
Responsibility for strategic plan	Weak	9	37.5	55.74	12.27
	Moderate	14	58.3		
	Good	1	4.2		
	
Documenting the strategic plan	Weak	2	8.3	62.22	10.85
	Moderate	20	83.3		
	Good	2	8.3		
	
Implementing the strategic plan	Weak	12	50.0	53.71	17.08
	Moderate	11	45.8		
	Good	1	4.2		
	
Evaluating the strategic plan	Weak	6	25.0	59.94	17.94
	Moderate	14	58.3		
	Good	4	16.7		
	
Benefits of the strategic planning	Weak	3	12.5	65.15	14.20
	Moderate	17	70.8		
	Good	4	16.7		

**Table 4:** Relationship between strategic planning (SP) requirements and facilitators with planning, implementation, and evaluation phases

Variables		Planning phase			Implementation phase			Evaluation phase			Totally		
		M±SD	F	Sig	M±SD	F	Sig	M±SD	F	Sig	M±SD	F	Sig
	
Employing consultant	No	60.3±12.5	.97	.33	54.3±17.5	.00	.97	56.6±15.0	1.2	.27	58.7±11.9	.00	.94
	Yes	63.5±9.7			53.2±17.4			62.2±19.9			61.8±10.9		
	
Employing ISO 9001	No	60.8±10.3	.23	.63	50.7±15.7	.32	.57	59.8±14.8	2.8	**.10**	59.1±10.2	1.9	.18
	Yes	65.5±12.0			60.8±19.2			60.1±25.3			63.9±13.5		
	
Employing EFQM	No	60.1±10.5	.00	.94	51.0±15.4	.33	.56	57.3±16.8	.12	.73	58.2±10.6	.18	.67
	Yes	66.4±10.9			59.0±19.9			65.1±20.1			65.1±11.7		
	
Having a SP committee	No	59.0±4.1	2.9	**.09**	41.9±21.1	.16	.68	47.0±14.4	.27	.60	54.4±5.3	2.8	.10
	Yes	62.8±11.7			56.0±15.7			62.5±17.7			61.7±11.8		
	
Active SP committee	No	56.9±9.0	.05	.81	54.1±19.9	2.4	.13	55.8±18.5	.00	.98	56.3±10.3	.13	.71
	Yes	67.5±10.1			53.3±14.5			64.0±17.1			64.8±10.8		
	
Having revision in SP	No	57.9±5.2	2.2	.14	47.0±14.9	1.0	.31	53.8±22.7	.86	.36	55.6±8.4	1.1	.29
	Yes	63.3±11.7			55.4±17.5			61.5±16.8			61.8±11.7		
	
Incentive for developing of SP	No	60.2±9.6	.64	.43	49.4±15.3	.47	.49	54.3±13.5	4.4	**.04**	57.6±9.1	2.7	.11
	Yes	65.5±12.4			60.8±18.3			69.3±21.0			65.5±13.1		
	
Having a documented SP	No	58.6±9.2	1.3	.26	47.9±12.2	5.1	**.03**	58.3±15.4	.99	.32	57.0±7.7	2.9	.10
	Yes	65.2±11.5			58.5±19.4			61.3±20.3			63.5±12.5		

### 2.4 Ethical Issues

This study was approved by Ethics Committee of Iran University of Medical Sciences Research. The main ethical issue was respondents’ right to self-determination, anonymity and confidentiality. The questionnaires were distributed among participants with a participant information sheet, explaining the nature of study. The consent was taken verbally from the participants. The questionnaire data were kept confidential and the respondents were assured of their right to withdraw at any time. The respondents’ names were not recorded on the questionnaire; thus, the data were rendered anonymously.

## 3. Results

All the studied hospitals were medical and teaching as national referral hospitals in Tehran providing medical and educational services. More than two-thirds of the hospitals were specialized and most of them had one evaluation degree. Establishment age of most of the hospitals was more than 30, half of them had more than 200 beds and about one-third had more than 500 employees. Most hospital managers had a Master’s degree in health services management.

From all the surveyed hospitals, about one-third had carried out the quality management system i.e. the International Organization for Standardize (ISO 9000) and European Fundamental for Quality Management (EFQM). All the hospitals are currently implementing national programs of accreditation and clinical governance requirements. The Balanced Scorecard method (BSC) was not considered in 15 hospitals (62.5%), while it is running in 9 hospitals (37.5%). More than half of the hospitals had a consultant for strategic planning. The majority of hospitals had a strategic planning committee; that is more than half of them have an active committee with monthly meeting. In more than half of the hospitals, members of strategic planning committee are between 5 to 9 persons.

All the hospitals have a documented strategic plan and in the majority of them, plan review was carried out. Most hospitals developed the strategic plan as a legal requirement of the Ministry of Health, while a limited number introduced improvement in performance and future prediction as an incentive for developing strategic plan.

In relation to the requirements and facilitators of strategic planning, most hospitals (n=15) have a moderate status. About developing strategic plan, two distinct dimensions were studied. They were stakeholder participation in strategic planning with an average score of 55.7%, and attention to the strategic planning components with an average score of 62.2% (moderate). Implementation of strategic plan consisted of two components. They were budget allocation based on the strategic priorities and action based on the strategic plan. Status of the studied hospitals with an average score of 53.71% was much weaker than other areas and half of the hospitals were weak in this area. Regarding the evaluation of strategic plan, using indicators for objectives achievement was studied and the average score of 59.94% reflected moderate status of the studied hospitals.

The results of the statistical analysis indicate a significant correlation between the incentive for developing of strategic plan and evaluation of strategic plan (P<0.04); that is the hospitals with incentive such as performance improvement attempted to develop a strategic plan and evaluate strategic plan more effectively. Also, a significant correlation was observed between having a documented strategic plan and implementation of plan (P<0.03). Among other requirements and facilitators, the strategic planning process and planning, implementation and evaluation showed no significant statistical correlation.

## 4. Discussion

In the present study, status of the formulation, implementation and evaluation of strategic plan in all the teaching hospitals affiliated with two major Medical Universities in Iran was investigated. The results showed that all the studied hospitals had strategic plan as a requirement of the national accreditation system; however, the strategic management was not conceptualized in these centers. Consequently, strategy implementation and particular activities related to the strategic control is not seen systematically.

Ideally, the strategic planning process involves all levels of managerial and operational of organization. Undoubtedly, the board of trustees in every organization has the primary responsibility to carry out the strategic planning. Through establishing key policy targets, the board should play a critical role in complex and extensive planning process (Cleverley & Cameron, 2007). In the present study, influential stakeholders, including hospital chief as a representative of physicians’ community, staff of operational levels, external stakeholders, representatives of patient/donor, and the representative of university had very little participation in the formulation of strategic plan. Due to the changing environment of healthcare delivery, physicians not only required to learn strategic planning and be involved in formulating it, but also they should, by engaging actively in the implementation of programs, have an effective role in achieving the organizational objectives (Schwartz, & Pogge, 2000). The study of Khajavi et al. (2009) in Iran showed that physicians’ skills in strategic thinking and planning are in a low level (Khajavi, Vatankhah, Maleki, Barati, Alami, & Heyrani, 2012). Schwartz and Pogge believed that the acquiring such skills requires a systematic and ongoing training (Schwartz & Cohn, 2002).

Executive administrator, assistants, matron, supervisors of clinical and supportive departments had appropriate participation in the formulation of strategic plan. It seems essential that key informants at various levels of within and outside of the organization have effective and adequate participation in formulating a strategic plan. In the study by Saleh et al. (2013) at Lebanon hospitals, the extent of participation of doctors and broad in formulating strategic plan was determined as moderate. Also, in the Amer et al. study at Texas hospitals, the board involvement in formulating strategic plan was reported to be medium (Kaissi et al., 2008).

Based on the findings, attention to various elements of strategic planning was at medium and high levels. Making a vision/study to forecast the future, assess the strength and weakness points and check the threats and opportunities have attracted the highest attention. However, assessing the feasibility of proposed strategies, competitive differentiation, understanding the concerns and needs of stakeholders, clarifying the rules and regulations of organizations and understanding customers’ demands and community needs was considered the least.

The status of strategic plan implementation in the form of budget allocation based on the strategic priorities and doing action according to the strategic plan was poor. Accordingly, in most of the studied hospitals, the university did not review annual budget based on the hospital’s strategic objectives. Moreover, hospital managers and authorities did not request budget based on the strategic plan. There was relatively minor attempt to identify and attract new financial resources to achieve the hospital’s strategic objectives. This is largely associated with the allocation of governmental budgets to hospitals.

Although the administrators and departments authorities in the studied hospitals are responsible for implementation of certain goals associated with the strategic plan, annual evaluation is not practiced based on the achievement of strategic objectives in most of the hospitals, and annual reward in most of the hospitals is not adjusted based on the rate of participation of different wards/individuals in achieving the strategic objectives. In majority of the hospitals, the responsibility for implementing the strategic plan is not assigned to the hospital manager and the annual evaluation is not done based on the achievement of strategic objectives. Unfortunately, hospitals in Iran, only in the formulation of strategic plan efforts to be acted based on existing scientific models and frameworks and in connection with the implementation of the plan are treated merely as traditional. In evaluations of MOH on the hospital’s strategic plan, the implementation of the action plan is not considered significantly.

In most of the studied hospitals, the rates of goals and projects progress derived from the strategic plan are regularly evaluated, and indicators of goal achievement are reviewed at certain time intervals and when necessary. In some hospitals, cause and effect relationship and balance between the indicators are considered. Analysis of measured values obtained from the indicators is done in limited range. The comparison the measured values with previous values, specified standards, and values of similar hospitals rarely done. Reporting the indicator values to the university, the community and stakeholders as well as design and performing appropriate interventions for improvement of performance are limited. Benchmarking of goal achievement indicators to ensure the effectiveness of strategic projects is limited in other hospitals. In the Mihic et al. study (2012), it is also indicated that healthcare organizations in Serbia attempt to formulate a strategic plan in appropriate manner, however, the monitoring and control of strategies implementation are not based on an scientific method (Mihic, Obradovic, Todorovic, & Petrovic, 2012).

The present results indicate that in cases that hospitals have attempted to establish standards of ISO 9001, status of evaluation strategic plan is more appropriate because of regular and systematic processes and activities as well as observance of documentation principles. According to Rusjan and Alic (2010), where ever the BSC model has been established as the strategic management tool, employing the quality management system (ISO 9001 QMS), has a positive impact on the strategic objectives of organization in four perspectives including customer satisfaction, financial results, internal processes, and learning and growth, (Rusjan & Alic, 2010). Also, using of BSC model in combination with EFQM, as an organizational excellence model, can lead to improvement in developing and implementing strategies in the healthcare provider organizations (Groene, Brandt, Schmidt, & Moeller, 2009). Both of these models can be considered as quality management tools, in the same way that it proposed for Spanish hospitals as an integrated management approach (Tejedor, 2009). It is essential to these excellence and quality management systems to be integrated within the organization strategic management. This initiative can be an interesting issue for future studies in the field of strategic management in hospital systems.

Finally, most of the studied hospitals something such as focus of hospital instructions on strategic issues, formation of an authentic sense of the hospital missions in community, clear definition of hospital priorities, providing training and development opportunities for all employees and provide high quality services have brought up as most important benefits of strategic management. Stating these benefits is in the condition which the studied hospitals mainly have worked well in the strategic planning, while implementation and evaluation of strategies in these hospitals is seriously impaired.

## 5. Conclusion

MOH requires Iranian hospitals have documented strategic plan in order to obtain points of accreditation. Hence, a strategic plan leads to performance improvement and increase in quality of care in hospitals. This is supported by our finding that the majority of studied hospitals had documented strategic plan. However, levels of development, implementation and evaluation of plans are moderate. The movement toward excellence, success, and favorable results depends on progress in these fields. The inappropriate status of three dimensions of strategic management in the studied hospitals can be attributed to insufficient knowledge about the terminology and process of strategic management, shortage of time to participate, poor beliefs on strategic management, administrative structure characterized by aversion plan, elitism sub-culture, and serious defect in teamwork, distrust in the external environment, and the centralized structure of MOH in notification of the programs.

It seems for resolving these problems and acquiring the benefits of strategic management in hospital systems in middle income countries, such as Iran, and low income countries, the authorities should attempt in short and long terms levels. A basic action to reduce many of these problems is creating the culture of program-based strategizing the organization, started in the high levels of the Ministry of Health, and then be flooded within the hospital systems. In the short term, the potentials of the Hospitals’ Accreditation National Program can be employed, and provide sufficient and appropriate training on the concept and on the process of strategic management then provide appropriate financial and non-financial incentives, in order to move hospitals towards centralized planning and strategic thinking.

To resolve these problems, the focus of future studies on the identification of infrastructure required for strategizing hospitals and being successful in implementing strategic management system, and on identifying the potential barriers for deployment strategies and evaluating results is recommended.

## 6. Limitation

This study had some potential limitations that may affect the results. The study was limited to hospitals of two universities in a single city. Therefore, generalizability of the results can be considered as a limitation of the present study. The findings were solely results of a research study and possibly more specialized investigation of these hospitals for strategic planning process will show the most precise results. Another limitation of this study is lack of literature particularly at international level to compare the results. In comparison to other Iranian hospitals, the studied hospitals have a higher proportion of governmental ownership, which limits generalizations of results.
